# Exploring attentional focus of older adult fallers during heightened postural threat

**DOI:** 10.1007/s00426-019-01190-6

**Published:** 2019-05-22

**Authors:** Toby J. Ellmers, Adam J. Cocks, William R. Young

**Affiliations:** 1 College of Health and Life Sciences, Brunel University London, Uxbridge, UK; 2 Institute of Environment, Health and Societies, Brunel University London, Uxbridge, UK; 3 Centre for Cognitive Neuroscience, Brunel University London, Uxbridge, UK

## Abstract

**Objectives:**

Threats to balance, and subsequent increases in fall-related anxiety, can disrupt attentional processing during gait in older adults, leading to behavioral adaptations which may increase fall risk. However, limited research has investigated what changes in attention occur to contribute to these disruptions. The aim of this research was to describe changes in attention that occur during gait when older adults’ balance is threatened, while exploring how previous fall history and trait movement reinvestment (conscious monitoring and control of movement) also influence attention.

**Methods:**

Forty older adults reported where they focus their attention when walking during two scenarios: (1) when they are relaxed and there is little risk of falling, and; (2) when their balance is threatened and they are anxious of falling.

**Results:**

During the high-threat condition, participants reported greater attention towards movement processes, threats to balance, worries/disturbing thoughts and self-regulatory strategies, with less attention directed towards task-irrelevant thoughts. However, fall history influenced attentional focus, with fallers directing greater attention towards worries/disturbing thoughts. Contrary to predictions, trait movement reinvestment was not associated with attention directed towards movement processes.

**Discussion:**

As processing worries/disturbing thoughts will likely reduce attentional resources available for effective postural control, we highlight this as one potential area to target interventions aimed at reducing the likelihood of repeated falling.

**Electronic supplementary material:**

The online version of this article (10.1007/s00426-019-01190-6) contains supplementary material, which is available to authorized users.

## Introduction

It is widely accepted that anxiety can influence both cognition and behavior, inducing changes in attentional focus which may subsequently disrupt movement coordination (for overviews, see: Eysenck & Wilson, 2016; Nieuwenhuys & Oudejans, 2012). Empirical support for these conclusions is drawn largely from research evaluating the execution of ontogenetic skills, such as sporting movements during high-pressure situations (e.g., Beilock & Carr, 2001; Wilson, Vine, & Wood, 2009). However, anxiety’s negative effect on how we think and move is not just confined to sport. An emerging body of research demonstrates the detrimental effects that anxiety can also exert on daily activities, such as controlling posture and gait.

Fall-related anxiety, or fear of falling, has been shown to disrupt attentional processing during gait in older adults (Gage, Sleik, Polych, McKenzie, & Brown, 2003), leading to behavioral adaptations which may, paradoxically, increase the risk of falling (Young & Williams, 2015).^1^ Elderly falls are a major public health concern. They are the leading cause of injury, and mortality from injuries, in those aged 65 years and older (Centers for Disease Control and Prevention, 2016), and cost the US economy over $30 billion annually (Burns, Stevens, & Lee, 2016). As anxiety is related to a 53% increase in fall risk (Hallford, Nicholson, Sanders, & McCabe, 2017), identifying mechanisms through which anxiety may impair attentional processing, and subsequently reduce safety during gait, is of significant value.

Recent findings from the domain of posture and gait indicate that fall-related anxiety may impair attentional processing efficiency by virtue of individuals directing attentional focus internally, in an attempt to consciously control or monitor movement (Ellmers & Young, 2018; Huffman, Horslen, Carpenter, & Adkin, 2009; Young, Olonilua, Masters, Dimitriadis, & Williams, 2015; Zaback, Cleworth, Carpenter, & Adkin, 2015). These findings broadly support *self*-*focus* theories of anxiety-related performance breakdown (Beilock & Carr, 2001). For example, Reinvestment Theory (Masters & Maxwell, 2008)—one such self-focus theory—postulates that anxiety leads the performer to direct conscious attention towards monitoring or controlling previously ‘automatic’ movement processes. Adopting such an attentional strategy is argued to disrupt movement execution (Masters & Maxwell, 2008) which, in the context of older adults, may lead to behavioral adaptations which reduce safety when walking (Young & Williams, 2015). For example, consciously controlling movement has been suggested to contribute to postural ‘stiffening’ (Young & Williams, 2015), whereby individuals freeze the degrees of freedom in the kinematic chain, effectively serving to reduce movement amplitude and ‘fluency’. While this postural control strategy may be beneficial in accommodating destabilizing factors during static postural tasks (for example, maintaining stability when a bus goes over a speed bump), postural stiffening will likely increase the possibility of falling during dynamic tasks (such as walking along an uneven pavement), where co-ordinated, skilled, and sometimes rapid movements are required to maintain safety (Young & Williams, 2015).

Research also suggests that attempting to consciously process walking/stepping movements may impair movement planning. For example, adopting an internal focus of attention has been shown to reduce proactive visual search during adaptive gait, with individuals fixating on the ground one step ahead at the expense of previewing future stepping constraints approximately four steps ahead (Ellmers & Young, 2019). Consequently, Uiga, Capio, Wong, Wilson, and Masters (2015) propose that such internal focus may increase fall risk by increasing the likelihood that these individuals will miss external information necessary for successful locomotion.

Alternatively, rather than performance disruptions resulting from directing *too much* on-line attention towards movement execution (as hypothesized by self-focus accounts), *distraction* theories propose that anxiety disrupts performance as a result of directing *too little* attention towards movement. Specifically, these theories hold that anxious individuals will preferentially direct attention towards threatening, task-irrelevant cues, which reduces the attentional resources available for processing task-relevant information necessary for successful task performance (Wine, 1971). These stimuli can be either internal (e.g., worries or disturbing thoughts relating to task failure) or external (threatening task-irrelevant environmental distracters). Attentional Control Theory (ACT; Eysenck, Derakshan, Santos, & Calvo, 2007), however, posits that anxious individuals can overcome these distractions using compensatory self-regulatory strategies; however, doing so is cognitively taxing and further reduces cognitive resources available for directing attention towards the primary task. This could be particularly troublesome for older adults, given both the age-related decrease in working memory capacity (e.g., Schneider-Garces et al., 2010) and the age-related increase in the minimum level of cognitive input required to maintain postural stability (Boisgontier et al., 2013; Woollacott & Shumway-Cook, 2002). Therefore, processing worries related to falling—and the subsequent cognitively taxing self-regulatory strategies employed to overcome such ruminative thoughts—can be viewed as a separate, secondary task; in that doing so will further reduce the already limited cognitive resources available for postural control, thereby resulting in greater postural instability and compromised safety.

Despite these contrasting theoretical stances, little attempt has been made to investigate likely changes in attention that occur in older adults during anxious gait. Instead, the limited research which has studied anxiety-related changes in attention during postural tasks has, hitherto, restricted these investigations to healthy young adults during conditions of artificially manipulated fall-related anxiety (Ellmers & Young, 2019; Johnson, Zaback, Tokuno, Carpenter, & Adkin, 2019b; Zaback, Carpenter, & Adkin, 2016). As such, it cannot be assumed that observed results will generalize to older adults experiencing threats to their balance in a complex setting typical of daily life (e.g., traversing a set of uneven paving stones in a crowded street). Therefore, the primary aim of this present research was to investigate how heightened postural threat (and subsequent increases in fall-related anxiety) modifies older adults’ self-reported attentional allocation during locomotion in real-world settings.

The secondary aim was to identify how older adults at a high risk of falling, such as those who have previously fallen (Dionyssiotis, 2012; Nevitt, Cummings, & Hudes, 1991) or individuals with a propensity to consciously control or monitor their movements (Wong, Masters, Maxwell, & Abernethy, 2008; Wong, Masters, Maxwell, & Abernethy, 2009; Young et al., 2015), alter their allocation of attention when their balance is threatened. For example, as older adult fallers are more likely to experience fear of falling (Friedman, Munoz, West, Rubin, & Fried, 2002)—characterized by Tinetti and Powell (1993) as a lasting concern about falling—it is possible that these individuals will allocate greater attention towards worrisome thoughts about both their previous falls and possible future accidents, especially when their balance is threatened. Similarly, Reinvestment Theory (Masters & Maxwell, 2008) posits that individuals with a propensity to consciously control/monitor their movements will direct greater attention towards conscious movement processing when anxious.

Owing to difficulties (both experimentally and ethically) of inducing fall-related anxiety in older adults in a naturalistic setting, we employed retrospective methods in a manner similar to that described by Oudejans et al. when investigating anxiety-related changes in allocation of attention in athletes (Oudejans, Kuijpers, Kooijman, & Bakker, 2011) and musicians (Oudejans, Spitse, Kralt, & Bakker, 2017). In the present research, older adults were asked to describe their thoughts and attention during a scenario when there is a very high risk of falling and their anxiety is at a peak. This retrospective verbal reports approach has been highlighted as a viable method for exploring “thoughts and attention without explicitly manipulating attention” (Oudejans et al., 2011, p. 62). We predicted that older adults would direct greater attention towards both movement processing and threats to balance during high-threat situations, and less attention towards task-irrelevant thoughts. However, we also predicted that fallers would allocate additional attention towards worries related to falling, and self-regulatory strategies attempting to overcome such distractive ruminations. Finally, we predicted that a higher trait propensity to consciously control/monitor movements would be associated with greater attention directed towards movement processing during high-threat situations.

## Methods

### Participants

Forty community-dwelling older adults (aged > 60; female/male: 28/12; mean ± SD age 76.50 ± 8.84) were recruited from sheltered residential accommodation schemes and community exercise classes. Previous research investigating the influence of fall-related anxiety on attention has reported effect sizes (*r *= *Z*/√*N*) between 0.57 and 0.60 for key, comparable variables (Ellmers & Young, 2019). Consequently, a power analysis conducted with G*Power (Faul, Erdfelder, Buchner, & Lang, 2009) determined that between 19 and 21 participants would be required to obtain 80% power (Cohen, 1988).

All participants were free from any neurological impairment or musculoskeletal condition that prohibited them from walking in daily life. Participants were excluded if they demonstrated major cognitive impairment [MiniCog score of < 3 (Borson, Scanlan, Brush, Vitaliano, & Dokmak, 2000; Borson, Scanlan, Chen, & Ganguli, 2003; Borson, Scanlan, Watanabe, Tu, & Lessig, 2006)]. Institutional ethical approval was obtained from the local ethics committee and the research was carried out in accordance with the principals laid down by the Declaration of Helsinki. All participants provided written informed consent.

### Assessments

The Movement Specific Reinvestment Scale (MSRS; Masters, Eves, & Maxwell, 2005) was used to assess participants’ trait movement reinvestment (Masters & Maxwell, 2008). This 10-item questionnaire consists of two 5-item subscales: conscious motor processing (i.e., ‘movement control’; R-CMP) and movement self-consciousness (i.e., ‘movement monitoring’; R-MSC). Items are rated on a 6-point Likert scale (1 = *strongly disagree*; 6 = *strongly agree*). Both subscales range from 5 to 30, with higher scores reflecting a higher propensity for reinvestment.

The Falls Efficacy Scale-International (FES-I; Yardley et al., 2005) was used to assess participants’ balance confidence. The 16-item questionnaire measures the level of concern about falling during a range of different activities, both inside and outside the home. Items are rated on a 4-point Likert scale (1 = *not at all concerned*; 4 = *very concerned*). Scores range from 16 to 64, with higher scores reflecting greater concern relating to balance (i.e., lower levels of balance confidence). The questionnaire has been recommended as an appropriate screening tool for fall-related concern in both research and clinical settings (Delbaere, Close, Mikolaizak et al., 2010).

Cognitive functioning was assessed with the MiniCog (Borson et al., 2000). The MiniCog is a composite of delayed three-item recall and clock drawing (participants are instructed to draw a clock with the hands pointing to a specified time on a blank clock-face). The maximum score possible is 5, with 1 point assigned for every correctly recalled item and 2 points assigned for a correctly drawn clock. This assessment (when using a cutpoint of 3) has been demonstrated to have similar levels of sensitivity at detecting cognitive impairment as the Mini-Mental State Examination (Borson et al., 2003).

Physical functioning was assessed using the Timed Up and Go test (TU&G; Podsiadlo & Richardson, 1991). In this test, participants are timed while they stand up from a chair (approximate seat height 46 cm), walk 3 m at a comfortable and safe pace, turn around, return to the chair, and sit back down. The test is commonly used in both clinical and research settings as a means of assessing physical functioning (Gates, Smith, Fisher, & Lamb, 2008; Podsiadlo & Richardson, 1991). Data from these assessments are presented in Table 1.

**Table 1:** Participant characteristics

Measure: mean (± standard deviation)	Non-faller group (*n* = 22)	Faller group (*n* = 18)
Age	76.55 (± 9.14)	76.44 (± 8.99)
Gender (females)	13	15
Number of falls (past 12 months)	0	1.56 (± 1.25)
TU&G (s)	11.02 (± 5.52)	12.64 (± 4.89)
MiniCog	4.30 (± 0.80)	4.53 (± 0.64)
FES-I	27.41 (± 9.72)	32.94 (± 9.78)*
R-CMP	17.18 (± 7.69)	19.62 (± 6.78)
R-MSC	12.23 (± 6.87)	12.78 (± 8.18)

*p < 0.05

Participants were classified as fallers (one or more falls; *n* = 18) or non-fallers (zero falls; *n* = 22) based on the number of times they recalled falling in the past 12 months. A fall was defined as an event in which the individual unintentionally came to rest on the ground, floor, or another lower level (Koski, Luukinen, Laippala, & Kivela, 1996). Four fallers had experienced an injurious fall, resulting in hospitalization. However, all participants had been discharged from hospital and had returned to independent community living by the time of participation.

### Verbal reports procedure

Participants were asked to imagine themselves walking during two scenarios: low- and high-threat. For the low-threat condition, participants were presented with the following scenario:

Think about a moment during walking when you are completely relaxed and there is a low chance of tripping or falling. For example, you could be walking on a flat, even surface or walking in a familiar, safe environment.

Participants were then asked two questions to explore their attentional focus: “When you are completely relaxed, what do you think about and focus your attention to? What do you do to ensure that you do not trip or fall?” Participants were instructed to provide at least one answer for each question. For the high-threat condition, participants were presented with the following scenario:

Think about an important moment during walking, when your anxiety is very high and there is a very strong chance of tripping or falling if you do not execute the next step well. For example, you could be walking through a busy crowd, stepping off a high curb, or walking on a slippery (wet or icy) or uneven surface.

Participants were then asked two questions: “When your anxiety is at its peak, what do you think about and focus your attention to during these important moments? What do you do to try and prevent yourself from tripping or falling?” As with the low-threat condition, participants were instructed to provide at least one answer for each question. The order in which participants were presented the low- and high-threat scenarios was counterbalanced.

To ensure that participants could relate to the scenarios presented, the example scenarios provided were designed to feature activities that are frequently encountered by community-dwelling older adults (such as walking along a flat surface/uneven surface/through a crowd, etc.). Pilot testing further confirmed the suitability of these scenarios for a cohort of community-dwelling older adults. These scenarios and follow-up questions were derived from those previously used by Oudejans et al. (2011, 2017), with the second question similarly included to provide participants with an additional prompt. While it was hypothesized that this second question would be more relevant for the high-threat scenario, it was included for both conditions to allow us to establish a low-threat baseline against which we could compare threat-related behaviors. For example, while older adults may report that they control their movement and step carefully when their balance is threatened, it is possible that they may also display these behaviors when relaxed and there is a low perceived risk of falling. As per Zaback et al. (2016), a simple probe (“Can you please explain what you mean by this statement?”) was used in instances where answers provided required further clarification.

### Data analysis

Verbal reports were analyzed by two independent observers (authors TJE and AJC). As both questions served the same purpose, answers to each were combined and analyzed together (Ellmers & Young, 2019; Oudejans et al., 2011). Both observers produced a list of statements from the verbal reports. This process involved separating the verbal reports into single, codable statements (allowing each statement to be coded into a single category), as well as omitting any statement describing something towards which attentional focus could not be directed (Oudejans et al., 2011, 2017). The two statement lists of both observers showed a high inter-observer reliability of 93.6%. Any discrepancies between the two lists were discussed until agreements were reached, leading to a final list of 226 statements.

As with previous research (Ellmers & Young, 2019), each statement was categorized into one of the five following attentional categories:

1. Movement processes (thoughts relating to consciously controlling or monitoring movement, e.g., “I focus on picking up my feet” or “I focus on walking slowly”)
2. Threats to balance (thoughts about environmental threats to balance, e.g., an uneven paving stone or an approaching cyclist)
3. Worries or disturbing thoughts (e.g., thoughts relating to falling and the potential negative consequences of this)
4. Self-regulatory strategies (positive self-talk statements, as well as thoughts adopted to enhance concentration, e.g., “I concentrate on making my breathing more controlled”)
5. Task-irrelevant information (statements unrelated to walking or maintaining balance, e.g., an individual thinking about what they are having for dinner or letting one’s mind wander).

Statements were categorized by two observers (TJE and AJC) independently, resulting in 97.1% inter-observer reliability. Any disagreements were discussed until an agreement was met. Examples of categorized statements from the present study are presented in Table 2.

**Table 2:** Example items (coded statements from the present research) for each attentional category

Attentional categories	Examples
Movement processes	Participant 31: “Step more deliberately (control where I am stepping)” Participant 32: “I always focus on lifting my right foot to make sure it doesn’t catch on anything”
Threats to balance	Participant 8: “Even when I am relaxed while walking in the flat or along the road, park, anywhere, I keep my eyes down for potential threats” Participant 37: “Looking at the ground to make sure there is nothing to trip me up”
Worries or disturbing thoughts	Participant 2: “I fell outside the main door and spent 3 weeks in hospital with two fractures… I think about that every time I go out the door” Participant 35: “Thinking about falling and injuring myself. I’ve had some very nasty falls… I will never forget the times I fell down the marble stairs”
Self-regulatory strategies	Participant 38: “Tell myself to ‘come on and do it’ and continue despite the anxiety” Participant 40: “Thinking what might be causing anxiety so as to help me relax”
Task-irrelevant information	Participant 10: “General thoughts about plans for the day” Participant 11: “Often let my mind wander, what to have for dinner, who I need to contact, etc.”

### Statistical analysis

As all verbal report data were non-normally distributed, it was not possible to use multiple 2 × 2 (low/high-threat × faller/non-faller) ANOVAs to compare within- and between-group differences for each of the five attentional categories. Therefore, the statistical analyses were separated into three sections: (1) general changes in attentional focus; (2) faller vs. non-faller comparisons, and; (3) correlational analyses.

#### General changes in attentional focus

Wilcoxon tests were used to determine the low- to high-threat change in the number of verbal reports generated for each of the five attentional categories (Ellmers & Young, 2019; Zaback et al., 2016). As the assumption of normality was violated, effect size is reported as *r* = *Z*/√*N* (Fritz, Morris, & Richler, 2012).

#### Faller vs. non-faller comparisons

For each attentional category, two separate Mann–Whitney *U* tests were used to explore between-group (faller/non-faller) differences for the number of reports generated; one test for low- and one test for high-threat (Oudejans et al., 2017). Separate Wilcoxon tests for fallers and non-fallers were then used to determine within-group changes between low- and high-threat for each attentional category. Bonferroni was corrected to 0.0125 for all analyses, based on the four separate analyses conducted for each category: (1) between-group analysis comparing fallers vs. non-fallers at low-threat (Mann–Whitney *U* test); (2) between-group analysis comparing fallers vs. non-fallers at high-threat (Mann–Whitney *U* test); (3) within-subject change (low- to high-threat) for fallers (Wilcoxon test); (4) within-subject change (low- to high-threat) for non-fallers (Wilcoxon test). Effect size is reported as *r* = *Z*/√*N*.

#### Correlational analyses

Separate partial Spearman’s correlations were used to compare the relationships between participant characteristics (number of falls, R-CMP, R-MSC, FES-I) and the number of verbal reports generated for each attentional category (during both low- and high-threat), while controlling for the potential following confounds: age, TuG and MiniCog scores. Correlations were only completed on verbal report categories containing a minimum number of 20 statements (see Table 3). This decision was made to ensure that results were not confounded by conducting correlations on categories with, for example, only 5 items (e.g., movement processes or self-regulatory strategies during low-threat). This resulted in correlations being used to compare participant characteristics and the following attentional categories: threats to balance (low-threat); task-irrelevant information (low-threat); movement processes (high-threat); threats to balance (high-threat); worries or disturbing thoughts (high-threat), and; self-regulatory strategies (high-threat). Based on the highly correlated nature of numerous participant characteristics, in any instances where an attentional category was significantly correlated with two or more participant characteristics, seperate follow-up non-parametric partial correlations controlling for any other significantly correlated participant characteristic (in addition to age, TuG and MiniCog scores) were conducted. For example, if R-CMP and FES-I were both significantly correlated with movement processing statements, then two separate follow-up correlations would be conducted: one correlating R-CMP and number of movement processing statements, controlling for FES-I, age, TuG and MiniCog scores; and another correlating FES-I and number of movement processing statements, controlling for R-CMP, age, TuG and MiniCog scores).

**Table 3:** Number (and percentage) of statements in each attentional category, and the number (and percentage) of participants producing these statements, for both low- and high-threat

Attentional category	Low-threat		High-threat	
	Number of statements	Number of participants	Number of statements	Number of participants
Movement processes***	5 (5.5%)	5/40 (12.5%)	44 (32.6%)	30/40 (75.0%)
Threats to balance***	22 (24.2%)	17/40 (42.5%)	42 (31.1%)	29/40 (72.5%)
Worries or disturbing thoughts**	5 (5.5%)	3/40 (7.5%)	25 (18.5%)	17/40 (42.5%)
Self-regulatory strategies**	5 (5.5%)	5/40 (12.5%)	22 (16.3%)	17/40 (42.5%)
Task-irrelevant information***	54 (59.3%)	33/40 (82.5%)	2 (1.5%)	1/40 (2.5%)
Total	91 (100%)		135 (100%)	

**p < 0.01, ***p < 0.001 (when the number of statements produced was statistically compared between low- and high-threat)

## Results

### General changes in attentional focus

Attention directed towards movement processes was more often reported in conditions of high-threat compared to low-threat (*Z* = − 4.62, *p* < 0.001, *r* = 0.73). During high-threat, participants also reported directing more frequent attention towards threats to balance (*Z* = − 3.65, *p* < 0.001, *r* = 0.58), worries or disturbing thoughts (*Z* = − 3.44, *p* = 0.001, *r* = 0.54) and self-regulatory strategies (*Z* = − 2.50, *p* = 0.006, *r* = 0.40). They also reported directing significantly less attention towards task-irrelevant information (*Z* = − 5.30, *p* < 0.001, *r* = 0.84). These data are presented in Table 3.

### Faller vs. non-faller comparisons

#### Between-group differences

Compared to non-fallers, fallers reported directing significantly greater attention towards worries or disturbing thoughts during both low- (*U* = 154.00, *Z* = − 2.30, *p* = 0.011, *r* = 0.36) and high-threat (*U* = 63.50, *Z* = − 4.13, *p* < 0.001, *r* = 0.65). Fallers also reported significantly less attention directed towards task-irrelevant information during low-threat (*U* = 96.00, *Z* = − 3.02, *p* = 0.002, *r* = 0.48). No other between-group differences were found for faller status, *U*s ≥ 157.00, *Z*s ≤ − 1.27, *p*s ≥ 0.13, *r*s ≤ 0.36. These data are presented in Table 4.

**Table 4:** Number (and percentage) of statements in each attentional category, and the number (and percentage) of participants producing these statements, for non-fallers and fallers

Attentional category	Low-threat		High-threat	
	Number of statements	Number of participants	Number of statements	Number of participants
*Non*-*fallers*				
Movement processes***	3 (5.7%)	3/22 (13.6%)	27 (39.7%)	18/22 (81.8%)
Threats to balance***	8 (15.1%)	7/22 (31.8%)	24 (35.3%)	18/22 (81.8%)
Worries or disturbing thoughts	0 (0%)^†^	0/22 (0%)	3 (4.4%)^†††^	3/22 (13.6%)
Self-regulatory strategies	3 (5.7%)	3/22 (13.6%)	12 (17.7%)	8/22 (36.4%)
Task-irrelevant information***	39 (73.6%)^††^	21/22 (95.5%)	2 (2.9%)	1/22 (4.6%)
Total	53 (100%)		68 (100%)	
*Fallers*				
Movement processes**	2 (5.3%)	2/18 (11.1%)	17 (25.4%)	12/18 (66.7%)
Threats to balance	14 (36.8%)	10/18 (55.6%)	18 (26.9%)	11/18 (61.1%)
Worries or disturbing thoughts***	5 (13.2%)^†^	3/18 (16.7%)	22 (32.8%)^†††^	14/18 (77.8%)
Self-regulatory strategies	2 (5.3%)	2/18 (11.1%)	10 (14.9%)	9/18 (50.0%)
Task-irrelevant information***	15 (39.5%)^††^	12/18 (66.7%)	0 (0%)	0/18 (0%)
Total	38 (100%)		67 (100%)	

**p < 0.01, ***p ≤ 0.001 (when the number of statements produced was statistically compared between low- and high-threat, for both fallers and non-fallers—i.e., within-group comparisons)

^†^*p* < 0.0125, ^††^*p* < 0.01, ^†††^*p* < 0.001 (when fallers were statistically compared to non-fallers, for that respective condition—i.e., between-group comparisons)

#### Within-group changes (fallers)

Compared to during low-threat, fallers reported significantly more attention directed towards both movement processes (*Z* = − 2.71, *p* = 0.004, *r* = 0.64) and worries or disturbing thoughts (*Z* = − 3.03, *p* = 0.001, *r* = 0.71) during high-threat. They also directed significantly less attention towards task-irrelevant information (*Z* = − 3.45, *p* < 0.001, *r* = 0.81) during high-threat. No other differences were found when comparing changes between low- and high-threat for fallers, *Z*s ≤ − 2.00, *p*s ≥ 0.023, *r*s ≤ 0.47. These data are presented in Table 4.

#### Within-group changes (non-fallers)

During high-threat, non-fallers directed significantly more attention towards both movement processes (*Z* = − 3.82, *p* < 0.001, *r* = 0.81) and threats to balance (*Z* = − 3.35, *p* < 0.001, *r* = 0.71), and directed significantly less attention towards task-irrelevant information (*Z* = − 4.41, *p* < 0.001, *r* = 0.94). No other differences were found when comparing changes between low- and high-threat for non-fallers, *Z*s ≤ − 1.73, *p*s ≥ 0.042, *r*s ≤ 0.37. These data are presented in Table 4.

### Correlational analyses

Only significant correlations are reported in this section. Please see Table 5 for a complete list of *r*-values and *p*-values for all analyzed correlations. All correlations are reported while controlling for age, TuG and MiniCog scores.

**Table 5:** Relationships between participant characteristics and the number of verbal reports for attentional categories with a minimum of 20 statements

	Low-threat		High-threat			
	Threats to balance	Task-irrelevant information	Movement processes	Threats to balance	Worries or disturbing thoughts	Self-regulatory strategies
Number of falls						
*r*	0.250	− 0.472	− 0.073	− 0.145	0.703	0.036
*p*	0.080	0.003	0.344	0.210	< 0.001	0.421
FES-I						
*r*	0.230	− 0.471	0.167	0.076	0.191	− 0.111
*p*	0.099	0.003	0.177	0.338	0.143	0.268
R-CMP						
*r*	0.362	− 0.390	− 0.121	0.095	0.224	0.153
*p*	0.019	0.013	0.251	0.299	0.105	0.197
R-MSC						
*r*	0.482	− 0.034	− 0.090	− 0.026	0.022	0.164
*p*	0.002	0.425	0.310	0.444	0.451	0.181

#### Threats to balance (low-threat)

During low-threat, a significant positive association was observed between the number of statements related to threats to balance and both R-CMP (*r* = 0.36, *p* = 0.019) and R-MSC scores (*r* = 0.48, *p* = 0.002). However, when controlling for each significantly correlated participant characteristic, only R-MSC remained significantly correlated with low-threat task-irrelevant information (*r* = 0.39, *p* = 0.013).

#### Task-irrelevant information (low-threat)

During low-threat, a significant negative association was observed between the number of task-irrelevant information statements and: number of falls (*r* = − 0.47, *p* = 0.003); FES-I scores (i.e., greater fall-related concerns, *r* = − 0.47, *p* = 0.003), and; R-CMP scores (*r* = − 0.39, *p* = 0.013). However, when controlling for other significantly correlated participant characteristics, only number of falls remained significantly correlated with low-threat task-irrelevant information (*r* = − 0.41, *p* = 0.01).

#### Worries or disturbing thoughts (high-threat)

During high-threat, a higher number of falls were significantly correlated with the number of worries or disturbing thoughts reported (*r* = 0.70, *p* < 0.001).

## Discussion

The results demonstrate significant alterations in how older adults report directing their attention during scenarios where their balance is threatened and their anxiety about falling is high. The results also highlight marked differences in how individuals who have previously fallen allocate their attention. These findings extend previous research that had only investigated attentional changes during anxious gait in young adults (Ellmers & Young, 2019).

### General changes in attentional focus

As predicted, older adults reported directing greater attention towards both movement processes and threats to balance, and less attention towards task-irrelevant thoughts, when their balance was threatened (see Table 3). Increased attention towards the control and/or perception of movement when anxious supports Reinvestment Theory (Masters & Maxwell, 2008). In the present research, it is likely that increased attention was directed towards conscious movement processing in an attempt to minimize the likelihood of a fall occurring. However, as consciously controlling movement is associated with behavioral adaptations which may reduce safety during gait—such as postural ‘stiffening’ (Young & Williams, 2015) and disrupted movement planning (Ellmers & Young, 2019; Uiga et al., 2015)—this attentional strategy may, paradoxically, increase the likelihood that an individual will fall. Although, given the lack of significant between-group difference observed in the number of movement processing statements reported during high-threat scenarios—with both fallers and non-fallers reporting significantly more movement processing statements during high-threat—it is also possible that in some instances, such attempts to consciously control movement may serve a functional benefit (for example, if the individual possesses the cognitive resources required to simultaneously consciously process movement and plan future actions, or in instances where reductions in movement amplitude/fluency carry limited negative consequences). As such, we propose that it may be possible to view conscious movement processing as a behavioral trade-off between attempts to consciously negotiate an ongoing threat, and the negative consequences associated with either reductions in movement amplitude/fluency or disrupted movement planning. While traditional conceptualizations have viewed any attempts to consciously control/monitor dynamic gait-related tasks as a maladaptive process (e.g., Young & Williams, 2015), future work is needed to better understand the behavioral consequences of anxiety-related increases in conscious movement processing.

During high-threat, participants also reported directing greater attention towards both worries/disturbing thoughts, and self-regulatory strategies. These results support predictions made by ACT (Eysenck et al., 2007), which posits that anxiety may disrupt attentional processing as a result of directing preferential attention towards worries or disturbing thoughts. Processing these thoughts imposes not only “substantial demands on the processing and storage capacity of working memory…[but] an additional burden on the self-regulatory mechanism inhibiting such thoughts” (Eysenck et al., 2007, p. 337). Directing attention towards worries or disturbing thoughts, as well as the subsequent direction of attention towards self-regulatory strategies, will likely reduce the cognitive resources available for postural control and consequently reduce safety in this population, as older adults require increased cognitive input to effectively control posture and gait (Boisgontier et al., 2013; Woollacott & Shumway-Cook, 2002).

### Can observations in young adults be translated to older adult populations?

When completing an adaptive gait task under experimentally manipulated conditions of postural threat, Ellmers and Young (2019) reported that young adults similarly directed greater attention towards both movement processes and threats to balance, and less attention towards task-irrelevant thoughts. However, unlike the older adults studied in the present research, this young adult cohort did not report directing greater attention towards either worries or disturbing thoughts, or self-regulatory strategies. This suggests that differences may exist between how young and older adults allocate attention during gait when anxious about falling. Indeed, a large number of the worries or disturbing thoughts reported by the older adults in the present research were ruminations on previous falls (see Table 2). These results clearly demonstrate that personal experiences influence how attention is allocated during conditions of imagined postural threat.

It is also possible that these age-related differences may be partially attributed to differences in environmental context. For example, the older adults studied in the present research would have likely imagined a complex, challenging scenario during which there would have been a high chance of falling. In contrast, the young adults studied by Ellmers and Young (2019) would have likely viewed the laboratory-based experimental task utilized as representing both a lesser challenge and a smaller threat to balance. However, recent work conducted by Johnson, Zaback, Tokuno, Carpenter, & Adkin (2019a) highlights differences between how young and older adults allocate attention when performing an identical postural control task (i.e., no differences in environmental context). This suggests that differences in attentional allocation observed in the older adults in the present study, when compared to young adults in previous research (e.g., Ellmers & Young, 2019), are unlikely to primarily be a consequence of different environmental contexts. Instead, we suggest that these differences are more likely underpinned by differences in personal experience. As such, we suggest that one must be cautious when attempting to generalize work carried out in young adults to make inferences about how attentional allocation may compromise safety in older adults. While it may be possible to produce situations that induce fear of falling in young adults (e.g., Ellmers & Young, 2019; Zaback et al., 2016), due to the marked differences in previous personal experiences, it is unlikely that the subsequent attentional (and behavioral) response in this population will represent anxiety-related changes identical to those observed in older adults at risk of falling.

### Attentional focus during gait is dependent on previous fall experience

Our results demonstrate that attentional allocation, during both low- and high-threat, is dependent on previous personal experiences with falls. We observed significant differences in how elderly fallers allocate attention, compared to non-fallers (see Table 4). During low-threat, fallers directed greater attention towards worries/disturbing thoughts, and less attention towards task-irrelevant information, when compared to non-fallers. They also directed greater attention towards worries/disturbing thoughts during high-threat. In contrast, no such significant changes were observed in non-fallers. This indicates that the significant threat-related increase in the amount of attention directed towards worries or disturbing thoughts described in the present cohort, when analyzed as an overall group, is driven primarily by changes occurring within participants who have previously fallen.

These results further reinforce the dramatic adverse effects that falling can have for older adults. Previous research illustrates that falling can have a major negative influence on older adults’ quality of life, leading to both activity restriction and a loss of independence which extend beyond any consequences of physical injury resulting from the fall (Vellas, Wayne, Romero, Baumgartner, & Garry, 1997). Our present results extend these findings and highlight ruminative thoughts relating to the fall itself—thoughts which persist even during situations where the chance of falling is low—as one potential explanation as to why older adults who have previously fallen avoid even low-risk, everyday activities critical for independent living. Previous research has also highlighted a relationship between falling and the extent to which an older adult engages in thoughts unrelated to their behavioral goal [also termed ‘mind-wandering’ (Nagamatsu, Kam, Liu-Ambrose, Chan, & Handy, 2013)]. However, the content of these thoughts was not explored. Consequently, we suggest that these worries or disturbing thoughts observed during the present research represent one, potentially prevalent, form of mind-wandering, and may contribute to reduced safety while walking.

Interestingly, when the present cohort was analyzed as an overall group, we observed threat-related increases in attention directed towards threats to balance. However, these changes appear to be confined to the older adults who had not previously fallen, with no such significant changes observed in fallers. This was unexpected, as it is logical to assume that in the presence of increased threats to balance, individuals would direct greater attention towards such stimuli, to plan the postural adjustments necessary to ensure that the threat does not result in a loss of balance. However, it seems that older adults who have previously fallen may prioritize the processing of worries and disturbing thoughts (e.g., internal threats) at the expense of attending to the increased threats to their balance (e.g., external threats). While future research is needed to confirm this suggestion, failure to attend to relevant threats to balance when anxious will likely compromise safety by virtue of neglecting and failing to accommodate external information necessary for avoiding environmental hazards.

These findings indicate that during conditions of imagined postural threat, older adults who have not recently fallen will allocate attention in a manner similar to that previously described in healthy young adults during experimental conditions of increased postural threat (Ellmers & Young, 2019; Johnson et al., 2019b; Zaback et al., 2016). Specifically, they focus greater attention towards both identifying threats to their balance and subsequent attempts to consciously process movement, and less attention towards task-irrelevant thoughts. Given that these threat-related alterations in attention correspond to those previously reported in young adults, we propose that these changes may, in fact, reflect a protective/adaptive mechanism which enhances safety. For example, although conscious movement processing has been shown to disrupt movement planning (Ellmers & Young, 2019), perhaps far greater negative behavioral outcomes would occur if walkers prioritized the planning of future actions rather than allocating attention to immediate postural threats. Thus, it is possible that the negative behavioral outcomes associated with such mode of motor control (e.g., Young & Williams, 2015) become evident only in instances where conscious control/monitoring persists for longer than ‘necessary’ (e.g., beyond the navigation of a postural threat). Future work is needed to further explore this proposal.

In contrast, observed differences in how older adults—when analyzed as an overall group, compared to the young adults studied previously (Ellmers & Young, 2019; Johnson et al., 2019b; Zaback et al., 2016)—report attention during conditions of imagined postural threat appear to be driven largely by attentional changes reported by older adult fallers. We suggest that these differences are a likely consequence of previous (unsuccessful) experiences of encountering postural threats. For example, while the non-fallers likely imagined a previous situation where they successfully navigated a postural threat, fallers likely drew on a previous unsuccessful experience where they fell and subsequently attributed failure either internally (e.g., blaming their poor balance) or externally (e.g., blaming the poorly maintained pavement). Thus, while non-fallers report attentional responses that may have a protective benefit, we suggest that fallers would be more likely to report combinations of worries/disturbing thoughts and either movement processes (if attributing failure internally towards balance deficits) or threats to balance (if attributing failure externally towards environmental factors). While this speculative proposal indicates that fallers and non-fallers may have drawn upon different previous experiences when imagining scenarios of heightened postural threat, we do not view this as a confound. Instead, we suggest that—much like during other modes of motor control (e.g., highly pressured sport performance)—previous successful and unsuccessful threat-related experiences influence attentional allocation during subsequent threatening scenarios (real or imagined). As such, it is logical to assume that older adults who have fallen will ruminate about previous falls in instances where their balance is threatened—imagined, or real [much like athletes who have previously ‘choked’ under pressure will often ruminate on these previous failures during subsequent high-pressured situations (Hill, Hanton, Matthews, & Fleming, 2010)].

These results highlight that previous observations made regarding how young adults allocate attention when their balance is threatened may translate to cohorts of highly functioning older adults who have not recently fallen. However, the observed Faller/Non-faller differences also highlight the need to study high-risk older adults, such as those who have previously fallen, when attempting to make inferences about how changes in attention may influence fall risk.

### Trait movement reinvestment

Contrary to our predictions, we failed to observe an association between trait movement reinvestment and the amount of attention directed towards movement processes during high-threat. This was unexpected, as an individual’s propensity to consciously monitor and control their movements when anxious has been argued to be a dimension of personality (Masters & Maxwell, 2008). Research has demonstrated greater attention directed towards movement processing in older adults with higher levels of trait movement reinvestment (Uiga et al., 2015; Wong et al., 2009). However, this previous research did not investigate attentional focus during conditions of postural threat/anxiety. Therefore, it is possible that when fall-related anxiety is high and individuals are highly motivated to avoid a fall, older adults direct proportionate levels of attention towards movement processes regardless of their trait level of movement reinvestment. Higher levels of conscious movement processing have also been reported in older adults who have previously fallen (Wong et al., 2008, 2009). However, our results are contrary to these findings, with both older adult fallers and non-fallers reporting directing statistically comparable levels of attention towards movement processes during both low- and high-threat scenarios. While our present results do highlight the importance of considering state levels of conscious movement processing within the context of elderly falls, the predictions presented within Reinvestment Theory (Masters & Maxwell, 2008) regarding trait movement reinvestment—as measured through the MSRS (Masters et al., 2005)—appear to be less relevant within this context. This is an important issue, as previous research has concluded that the “MSRS [a measurement of trait movement reinvestment] shows potential as a clinical tool with which to predict falls in the elderly” (Wong et al., 2008, p. 410).

### Limitations

One limitation of the present research was the utilization of retrospective self-reports to investigate attentional focus. The aim of this study was to explore changes in attentional focus during ecologically valid situations of postural threat, thus avoiding potential confounds related to experimentally inducing fall-related anxiety (e.g., Ellmers & Young, 2019). Owing to the difficulties (both experimentally and ethically) of inducing fall-related anxiety in older adults in a naturalistic setting, we, thus, selected retrospective self-reports as the most appropriate methodology to answer our research question. While this method has been used previously to describe anxiety-related changes in attention in both athletes (Oudejans et al., 2011) and musicians (Oudejans et al., 2017), and is argued to be a viable method for exploring “thoughts and attention without explicitly manipulating attention” (Oudejans et al., 2011, p. 62), we are unable to determine the true extent to which retrospective self-reports reflect attentional allocation during daily life. This is particularly relevant for the present research, given the possibility that certain participants may have been unable to reliably recall and report attentional focus due to age-related cognitive decline. However, participants were excluded from participation if they demonstrated major cognitive impairment [MiniCog score of < 3 (Borson et al., 2000, 2003, 2006)]. Furthermore, the imagined scenarios were designed to feature frequently occurring, everyday experiences. Consequently, we reasoned that participants would have been able to relate to the imagined scenarios, resulting in accurate recollection and description of attentional allocation. Indeed, it is worth noting that our overall results are in line with those recently published by Johnson et al. (2019a), who found that older adults exposed to experimentally induced conditions of postural threat rated directing greater attention towards movement processes, threat-related stimuli (which included both external threats and internal worries) and self-regulatory strategies, and less attention directed towards task-irrelevant information.^2^ As such, despite the retrospective nature of the present research, we suggest that the findings presented by Johnson et al. (2019a) further highlight the validity of our data. Regardless, further research is needed which ethically manipulates fall-related anxiety in older adults during real-world scenarios, to evaluate attentional processes in ‘real time’.

Another limitation of utilizing retrospective self-reports relates to the possibility that the specific scenario imagined during both the low- and high-threat conditions differed between fallers and non-fallers. However, as we wanted the scenarios that participants generated to be individually meaningful and relevant, we reasoned that it would have been a greater confound to constrain participants to recall their attentional focus during a single, uniform scenario; a scenario which the participant may or may not have experienced. While we acknowledge that direct assessment of the specific scenarios which participants imagined may have provided further insight into the previously reported between-group differences, we deemed this unnecessary on the basis that the descriptions provided for the high-threat scenarios were designed to all include an external threat which needed to be navigated/avoided. As such, while the threat itself likely differed across participants, all imagined scenarios would have featured comparable opportunities for the individual to worry, consciously process movement, engage in self-regulatory strategies, and so on.

Finally, exploring attentional allocation through retrospective self-report does not allow for the investigation into how these changes in attention subsequently influence posture and gait-related behaviors. Future research should examine how fear of falling influences both the attention and behavior (for example, visual search behavior and stepping characteristics) of older adults during adaptive gait. The current findings can inform the most appropriate outcome measures and predictions in this future work.

## Conclusions

This study presents the first exploration of how older adults, specifically those at an increased risk of falling, reallocate attention when their balance is threatened during locomotion. The results support previous literature demonstrating that when their balance is threatened, like young adults (Ellmers & Young, 2019; Johnson et al., 2019b; Zaback et al., 2016), older adults are also less likely to direct attention towards task-irrelevant information, and more likely to focus on movement processes and threats to balance. However, these results also indicate that the amount of attention directed towards ruminative worries or disturbing thoughts is dependent on previous personal experiences with falling. Contrary to our predictions, trait movement reinvestment was not associated with the reporting of greater attention directed towards movement processes when threatened, indicating that the studying of state, rather than trait, movement reinvestment may be of more relevance for the context of elderly falls. As processing worries or disturbing thoughts will likely reduce the attentional resources available for effective postural control, thus compromising safety, we highlight this as one potential area in which to target interventions aimed at reducing the likelihood of repeated falling. Furthermore, as certain changes are dependent on previous personal experiences with falling, we suggest that one must be cautious when attempting to generalize work carried out in young adults to make inferences about fall risk in older adults. Given the subjective, retrospective nature of the measures assessed in the present research, objective ‘real-time’ measures of both attention and gait are needed to further explore these conclusions.

## Electronic supplementary material

Below is the link to the electronic supplementary material.

Supplementary material 1 (SAV 3 kb)

## Footnotes

1 While fear of falling may in some instances serve a protective effect (e.g., heightened fear may be a normal adaptive response to a realistic threat, which may prevent an individual with poor balance from undertaking an activity where there is a high chance of falling), older adults frequently display disproportionate levels of fear relative to their physiological fall risk (Delbaere, Close, Brodaty, Sachdev, & Lord, 2010). Excessive fear is associated with both behavioral responses to postural threats likely to reduce stability (Delbaere, Sturnieks, Crombez, & Lord, 2009), and an increased risk of falling (Delbaere et al., 2010). Indeed, as Delbaere et al. (2009) describe, “…when concern about falls is excessive, the associated adaptive behaviors might actually increase falls risk, rather than protect against it” (p. 241).

2 Note, Johnson et al. (2019a) did not separate participants into fallers/non-fallers. As such, a further sub-group comparison to our faller/non-faller data is not possible.
